# Mathematical models for predicting human mobility in the context of infectious disease spread: introducing the impedance model

**DOI:** 10.1186/s12942-017-0115-7

**Published:** 2017-11-22

**Authors:** Kankoé Sallah, Roch Giorgi, Linus Bengtsson, Xin Lu, Erik Wetter, Paul Adrien, Stanislas Rebaudet, Renaud Piarroux, Jean Gaudart

**Affiliations:** 1 INSERM, IRD, SESSTIM, Sciences Economiques & Sociales de la Santé & Traitement de l’Information Médicale, Aix Marseille Univ, Marseille, France; 2 Prospective et Coopération, Laboratoire d’Idées, Bureau d’Etudes Recherche, Marseille, France; 3 Service Biostatistique et Technologies de l’Information et de la Communication, APHM, Hôpital de la Timone, Marseille, France; 4 Department of Public Health Sciences, Karolinska Institutet, Stockholm, Sweden; 5 Flowminder Foundation, Stockholm, Sweden; 6 College of Information System and Management, National University of Defense Technology, Changsha, China; 7 Stockholm School of Economics, Stockholm, Sweden; 8 DELR, Ministère de la Santé Publique et de la Population, Port-au-Prince, Haiti; 9 APHM, Direction de la Recherche et de l’Innovation, Marseille, France; 10 UMR S 1136 INSERM, UPMC, Institut Pierre Louis d’Epidémiologie et de Santé Publique, Paris, France

**Keywords:** Human mobility, Disease spread, Impedance model, Spatial statistics, Epidemiology, Radiation model, Gravity model

## Abstract

**Background:**

Mathematical models of human mobility have demonstrated a great potential for infectious disease epidemiology in contexts of data scarcity. While the commonly used gravity model involves parameter tuning and is thus difficult to implement without reference data, the more recent radiation model based on population densities is parameter-free, but biased. In this study we introduce the new impedance model, by analogy with electricity. Previous research has compared models on the basis of a few specific available spatial patterns. In this study, we use a systematic simulation-based approach to assess the performances.

**Methods:**

Five hundred spatial patterns were generated using various area sizes and location coordinates. Model performances were evaluated based on these patterns. For simulated data, comparison measures were average root mean square error (aRMSE) and bias criteria. Modeling of the 2010 Haiti cholera epidemic with a basic susceptible–infected–recovered (SIR) framework allowed an empirical evaluation through assessing the goodness-of-fit of the observed epidemic curve.

**Results:**

The new, parameter-free impedance model outperformed previous models on simulated data according to average aRMSE and bias criteria. The impedance model achieved better performances with heterogeneous population densities and small destination populations. As a proof of concept, the basic compartmental SIR framework was used to confirm the results obtained with the impedance model in predicting the spread of cholera in Haiti in 2010.

**Conclusions:**

The proposed new impedance model provides accurate estimations of human mobility, especially when the population distribution is highly heterogeneous. This model can therefore help to achieve more accurate predictions of disease spread in the context of an epidemic.

**Electronic supplementary material:**

The online version of this article (10.1186/s12942-017-0115-7) contains supplementary material, which is available to authorized users.

## Background

Epidemic spread depends on the likelihood of infection, as well as on individual human interactions. Concerning the latter, mobility networks play a huge role in the temporal and spatial dynamics of disease transmission within a population [1].

If mobility networks cannot be provided, reaction diffusion models can roughly report on the epidemic spread [2]. In the last decade, there has been a growing interest among infectious disease epidemiologists in estimating human mobility and rebuild mobility networks [3]. While mathematical models of human mobility have been in use since the last century, they are of less value when real mobility data or reliable proxies—such as the call detail records (CDRs) of mobile network operators—are available [4]. The usefulness of these models appears in data-scarce contexts, such as during infectious disease epidemics in low-income countries, when forecasting the best possible allocation of resources becomes necessary. Indeed, these models can predict mobility patterns based solely on population size, population density, and travel distance.

The gravity and radiation models are the most commonly used mobility models today. The gravity model posits that mobility between two locations increases with population size and decreases with distance [5], whereas the radiation model assumes mobility to depend on population density [6]. Insofar as it relies on parameter tuning, the gravity model provides a broad theoretical framework that is pragmatically useless in the absence of specific space-dependent fitting [7]. As for the radiation model, it merely serves to predict the relative probability of mobility from a given location to different destinations—though it can help deduce absolute number of trips, provided that the average number of trips from each source location is known or approximated. In short, in data-scarce contexts such as in low-income countries, mathematical models of human mobility can only be used with minimal assumptions about the overall probability of mobility in the population.

The aim of this study was to introduce a new, parameter-free model, the impedance model, for predicting human mobility in the context of data scarcity.

In this article, we define the mobility models, translate them into formulations that allow for rigorous comparison, simulate a set of spatial patterns linked to various scenarios, and then compare the performance of the new impedance model, intuitively adapted from the laws of electricity, to previous well known models, for each simulated pattern. Lastly, we evaluate the performance of each of these models in predicting a real infectious disease spread, namely the 2010 cholera epidemic in Haiti.

## Methods

### Definitions

#### The impedance model

In order to estimate probabilities of mobility in data-scarce contexts, we proposed an intuitive and parameter-free model adapted from Ohm’s law of electricity (1827). We developed this model by simple analogy with the electric current model, where electric potential is represented by $P = I \times R$, *R* is the electric resistance, and *I* is the electric current (charge per unit of time). In a human mobility model, electric resistance is assimilated to distance (*d*), electric current to number of trips per day ($F_{ij}$), and electric potential to mobility potential per day on a given trajectory. The latter depends on the overall probability of mobility *α* applied to the size of the source and destination populations. It can be formulated as $\alpha \left( {P_{i} + P_{j} } \right)$.

Thus, according to the impedance model, the number of trips flow from *i* to *j* can be formulated as $F_{ij} = \alpha \frac{{P_{i} + P_{j} }}{{d_{ij} }}$.

The probability for a person in location *i* to travel from *i* to *j* is given by

1 $$\pi_{ij} = \frac{{F_{ij} }}{{T_{i} }} = \frac{{\alpha \frac{{P_{i} + P_{j} }}{{d_{ij} }}}}{{\sum\nolimits_{i \ne j} {\left( {\alpha \frac{{P_{i} + P_{j} }}{{d_{ij} }}} \right)} }} = \frac{{\frac{{P_{i} + P_{j} }}{{d_{ij} }}}}{{\sum\nolimits_{i \ne j} {\left( {\frac{{P_{i} + P_{j} }}{{d_{ij} }}} \right)} }}$$

This formula is parameter-free.

The previously known mobility models have also been developed by analogy with the laws of physical science. Among these, some have been pragmatically successful, and have thus become especially popular.

#### The gravity model

The gravity model estimates the number of trips *F*_*ij*_, between two geographical locations *i* and *j*, knowing their population sizes *P*_*i*_ and *P*_*j*_ and the distance between them, *d*_*ij*_, as

2 $$F_{ij} = k\frac{{P_{i}^{n} P_{j}^{m} }}{{f_{\gamma } (d_{ij} )}}$$

To allow rigorous comparisons, the formulation was adapted. See Additional file 1: A1

#### The radiation model

The radiation model was developed by analogy with the processes of emission and absorption of electromagnetic particles studied by physical scientists. The probability of commuting from *i* to *j* is given by

3 $$\pi_{ij} = \frac{{P_{i} P_{j} }}{{\left( {P_{i} + S_{ij} } \right)\left( {P_{i} + P_{j} + S_{ij} } \right)}}$$

More details on radiation model are given in Additional file 1: A2

### Simulations

#### Data generation

In order to assess the performance of our proposed model in plausible epidemiological situations, we generated data to build spatial patterns that could stand for real epidemic spaces. Location coordinates were generated using the double-uniform distribution. Each spatial pattern formed a square whose sides spanned between 1° and 40° in a polar coordinate system, and included a limited number of locations representing different demographic units. The area of each generated pattern varied approximately from 12,100 km^2^ (roughly the size of Gambia or Lebanon) to 19,360,000 km^2^ (roughly the size of Russia or North America). The number of generated locations in each spatial pattern varied randomly from 5 to 30. Each pattern reflected population densities that can be plausibly observed by demographers (100–10,000 inhabitants/km^2^). Two topological rules were applied to create plausible patterns: (1) each pattern had to include at least 10 km between locations of more than 100,000 inhabitants; and (2) the size of the population generated for each pattern had to be proportional to the pattern area.

Our aim was to account for geographical scale and to avoid the inconsistencies that come with considering huge populations in small areas (e.g., locations with more than 10,000,000 inhabitants in Gambia) or with examining very small populations in large areas (e.g., accounting for villages with small populations when studying disease spread throughout Russia).

The double-uniform distribution was used to generate population sizes. Population size heterogeneity was controlled by varying the gap between the two uniform distributions. In sensitivity analyses, the hypothesis used to generate population sizes was modified with a Poisson distribution, a double Poisson distribution, a normal distribution, and a truncated normal distribution. The regular grid pattern was also assessed to estimate result variability due to travel distances.

#### Simulation design

Reference data were needed to evaluate the performance of the three models on simulated patterns. In the absence of real mobility data or reliable proxies, we had to make assumptions to generate reference mobility data. Several scenarios were proposed, all of which had as a prerequisite a power deterrence function.

The first scenario—the source population and distance deterrence (SPDD)—assumed that the probability of mobility from source *i* to destination *j* decreased with the distance between *i* and *j* and was proportional to the size of the source population.

4 $$\pi_{ij} \propto P_{i} \times \frac{1}{{d_{ij} }}$$

The second scenario—the small to large population with distance deterrence (SLDD)— assumed that the probability of mobility on a given trajectory was proportional to the destination/source population size ratio and inversely proportional to distance.

5 $$\pi_{ij} \propto \frac{P_{j} }{P_{i} } \times \frac{1}{d_{ij} }$$

The third scenario—large to small population with distance deterrence (LSDD)—assumed that the probability of mobility was proportional to the destination/source population size ratio, while retaining the power deterrence function.

6 $$\pi_{ij} \propto \frac{{P_{i} }}{{P_{j} }} \times \frac{1}{{d_{ij} }}$$

The total number of trips from location *i* ($T_{i}$) depended on the overall probability of mobility (*α*) and on the size of the source population ($P_{i}$) (Eq. 8). The expected number of trips on a given trajectory *ij* ($F_{ij}$) followed a Poisson distribution (*P*), whose average depended on the probability of mobility from location *i* to destination *j* ($\pi_{ij}$) and on the total number of trips from location *i* (Eq. 8).

7 $$T_{i} = \alpha \times P_{i}$$

8 $$F_{ij} \sim P(\pi_{ij} \times T_{i} )$$

where *P* represented a Poisson distribution.

#### Number of simulations

The parameter to be estimated, $\alpha$, was the overall probability of mobility in the population. Probabilities of mobility estimated from various CDRs ranged from 1 to 5% (France, 2007: 0.026 [8], Kenya, 2012: 0.026 [9], Haiti, 2010: 0.035 [10], Spain, 2007: 0.011 [8], United States, 2011: 0.022 [6]). We used the range’s maximum value (5%) in the data generation process to approximate the worst-case conditions for infectious disease spread.

The epidemiological literature on disease spread does not provide recommendations on the threshold proportion of infected individuals that needs to be detected to avoid disease propagation. Obviously, this number depends on specific spatiotemporal patterns. Nevertheless, for methodological reasons, we assumed that a 1% error (*Δ*) in the estimated overall probability of mobility has significant consequences for emergency planning. This means that surveillance systems should be able to detect a variation in the overall daily probability of mobility corresponding to 100 persons moving from a small town of 10,000 inhabitants or to 100,000 persons moving from a huge agglomeration of about 10,000,000 inhabitants such as Paris. Assuming the maximum overall probability of mobility to be 10%, the maximum variance in the probability of mobility is σ^2^ = 0.1 * 0.1 = 0.01. According to simulation guidelines [11], the number of simulations needed to detect significant difference in the overall probability of mobility estimated by various models is given by

9 $$N = \left( {\frac{{Z_{1 - (\alpha /2)} \sigma }}{\Delta }} \right)^{2} = \left( {\frac{1.96 \times 0.1}{0.01}} \right)^{2} = 385\quad{\text{for}}\;\alpha \; = \;5\% \;{\text{significance}}\;{\text{level}}$$

For each scenario, we performed 500 independent simulations. The same spatial patterns were used to compare the three mobility models so that bias due to sample variability was eliminated while assessing the differences between the models.

#### Measures of performance

Statistical measures of performance were: overall probability of mobility $\hat{\alpha }$; bias in the probability of mobility ($\delta$); and average root mean square error (aRMSE) in number of trips.

The overall probability of mobility $\hat{\alpha }$ estimated by the model for a given pattern was defined as the percentage of travelers in the pattern’s population. This parameter can reveal over/underestimations of the overall probability of mobility, and is equal to

10 $$\hat{\alpha }=\frac{{\sum\nolimits_{i \ne j} {F_{ij} } }}{{\sum\nolimits_{i = 1}^{n} {P_{i} } }}$$

where *F*_*ij*_ is the number of trips on each *ij* trajectory estimated by the model for a given pattern, and *P*_*i*_ is the size of the population in the source location *n* of that pattern.

The bias *δ* associated with each model was defined as $\delta = \bar{\hat{\alpha }} - \alpha_{0}$, where $\hat{\alpha }$ is the overall probability of mobility estimated from a single simulation, $\bar{\hat{\alpha }}$ is the average rate over a set of *N* simulations, and *α*_0_ is the value of the overall probability of mobility used for data generation.

The *aRMSE* in number of trips estimates was computed for each mathematical model as the average of root mean square errors over a set of *N* simulations.

11 $$aRMSE = average\left( {\sqrt {\frac{{\sum\nolimits_{n} {\left( {F_{\text{model}} - F_{\text{ref}} } \right)^{2} } }}{n}} } \right)$$

where *F*_model_ and *F*_ref_ are the numbers of trips on a given trajectory—as estimated with model and reference data, respectively—*n* is the total number of trajectories in a given pattern, and *N* is the number of simulations for which the measure was computed.

### Modeling mobility patterns in the 2010 cholera spread in Haiti with a basic susceptible–infected–recovered (SIR) transmission framework

As a proof of concept, we used the data routinely collected during the first weeks of the cholera epidemic in Haiti by the Ministry of Public Health and Population. These data have already been used for the epidemiological analysis of the first year of the epidemic [12]. This epidemic, of exceptional magnitude, struck Haiti in October 2010, following the massive contamination of the Artibonite River after cholera was introduced in the country by a military contingent [13].

Briefly, morbidity data were prospectively collected at the commune level according to the World Health Organization standard definition [14].

In the current study we only analyzed data from October 27, 2010 to December 27, 2010, corresponding to the expansion phase of the epidemic, discarding the first 2 weeks a period when cholera transmission was related to the massive contamination of the river rather than a human-driven diffusion [13]. These early stages of the epidemic were modeled using a basic SIR framework.

We used two spatial definitions (*n* = 140 and *n* = 78 locations): The first corresponded to municipalities, and the second to agglomerations connected by a human mobility network [4]. The set of differential equations below represents the dynamics of transmission in each location *i*. The overall population was assumed to be at demographic equilibrium, with the birth rate balancing the mortality rate; it was also assumed to be not immune in the early stages of the epidemic.

12 $$\begin{aligned} \frac{{dS_{i} }}{dt} & = - \beta S_{i} (t)\left[ {\left( {1 - \alpha } \right)I_{i} (t) + \alpha \sum\limits_{j \ne i} {\pi_{ij} I_{j} (t)} } \right] \\ \frac{{dI_{i} }}{dt} & = \beta S_{i} (t)\left[ {\left( {1 - \alpha } \right)I_{i} (t) + \alpha \sum\limits_{j \ne i} {\pi_{ij} I_{j} (t)} } \right] - \gamma I_{i} \\ \frac{{dR_{i} }}{dt} & = \gamma I_{i} \\ \end{aligned}$$

The equations above represent the weekly variations in the number of individuals in the susceptible (*S*_*i*_), infected (*I*_*i*_) and recovered (*R*_*i*_) compartments at location *i*. *β* represents the rate of exposure to the disease—i.e., the rate of individuals becoming infected due to exposure to the effective incidence of cholera. The effective incidence of cholera in a location $i,\;(1 - \alpha )I_{i} (t) + \alpha \sum\nolimits_{j \ne i} {\pi_{ij} I_{j} (t)}$ was modeled as a weighted average of incidence rates in local and remote locations. Weights depended on the overall probability of mobility (*α*), but also on the relative probabilities of mobility from destination locations *j* to source location *i* which were provided by the matrices derived from CDRs or from the mathematical (impedance, gravity, radiation) models formulated in Eqs. 1, 3, and 4. *γ* is the recovery rate of the infected compartment. We evaluated mobility matrices under two hypotheses. The first made no assumption regarding specific overall probabilities of mobility from each location, allowing the rate *α* to be adjusted. The second assumed the overall probability of mobility from each location to be approximated by CDRs.

This CDRs mobility matrix resulted from the processing of cell phone network metadata of 2.9 million subscribers of the largest mobile phone operator in Haiti called Digicel (60% market share). Voice call and written messages metadata were collected daily from October 15 to December 19, 2010 [4, 10, 15]. The fraction of Subscriber Identity Module Cards (SIM cards) on the total population in 2013 was 61%. The maximum distance between two pairs of telephone towers was 38.6 km. The trajectories of subscribers between the phone towers made it possible to reconstitute the mobility network between geographical locations. For each pair of sites (*i*, *j*), the proportion of SIM cards in location *i* at time *t*, which were found at location *j* at time *t* + 1, was calculated.

To determine which model best describes the early stages of the cholera outbreak in Haiti, we compared the performances of the candidate models according to Akaike’s information criterion [16, 17] (AIC).

13 $$AIC = 2\theta + \eta \ln \left( {\frac{RSS}{\eta }} \right)$$

where *θ* is the number of estimated parameters in the model, and $\eta = n_{u} \times n_{w}$ is the number of data points (with $n_{u}$ and $n_{w}$ being the number of spatial units and the number of weeks since the start of the calibration period, respectively). The sum of the squares of the residuals between model estimates and epidemiological records is denoted by RSS.

14 $$RSS = \sum\limits_{u}^{{n_{u} }} {\sum\limits_{w}^{{n_{w} }} {\left[ {C(i,j) - \hat{C}(i,j)} \right]} }^{2}$$

where *C*(*i*, *j*) and $\hat{C}\left( {i,j} \right)$ correspond to the weekly number of reported cases and to the number of cases estimated by the model in a spatial unit *u* and a week *w,* respectively.

## Results

### Simulation results

#### Average root mean square error (aRMSE)

**Fig. 1:**
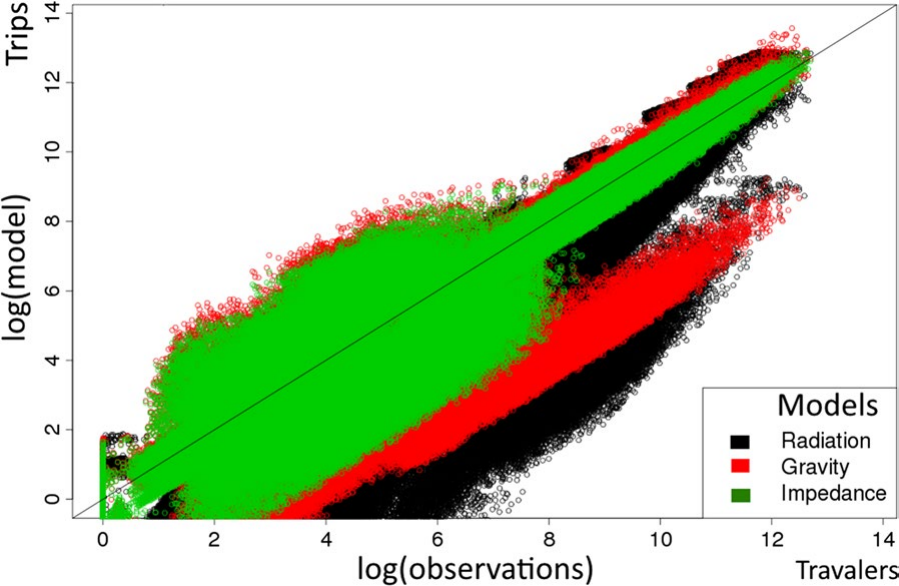
Number of trips estimated by the three models versus simulated reference data. Each dot represents the logarithm of the total number of trips on a given trajectory. The three simulation scenarios are combined for each model

Estimates of absolute flows from all simulations and all scenarios are shown in Fig. 1. The average root mean square error was lower with the impedance model (log(aRMSE) = 7.19 CI (7.10–7.35)) as compared with the gravity and radiation models (log(aRMSE) = 7.44 CI (7.34–7.54) and 8.40 CI (7.91–8.67), respectively).

**Fig. 2:**
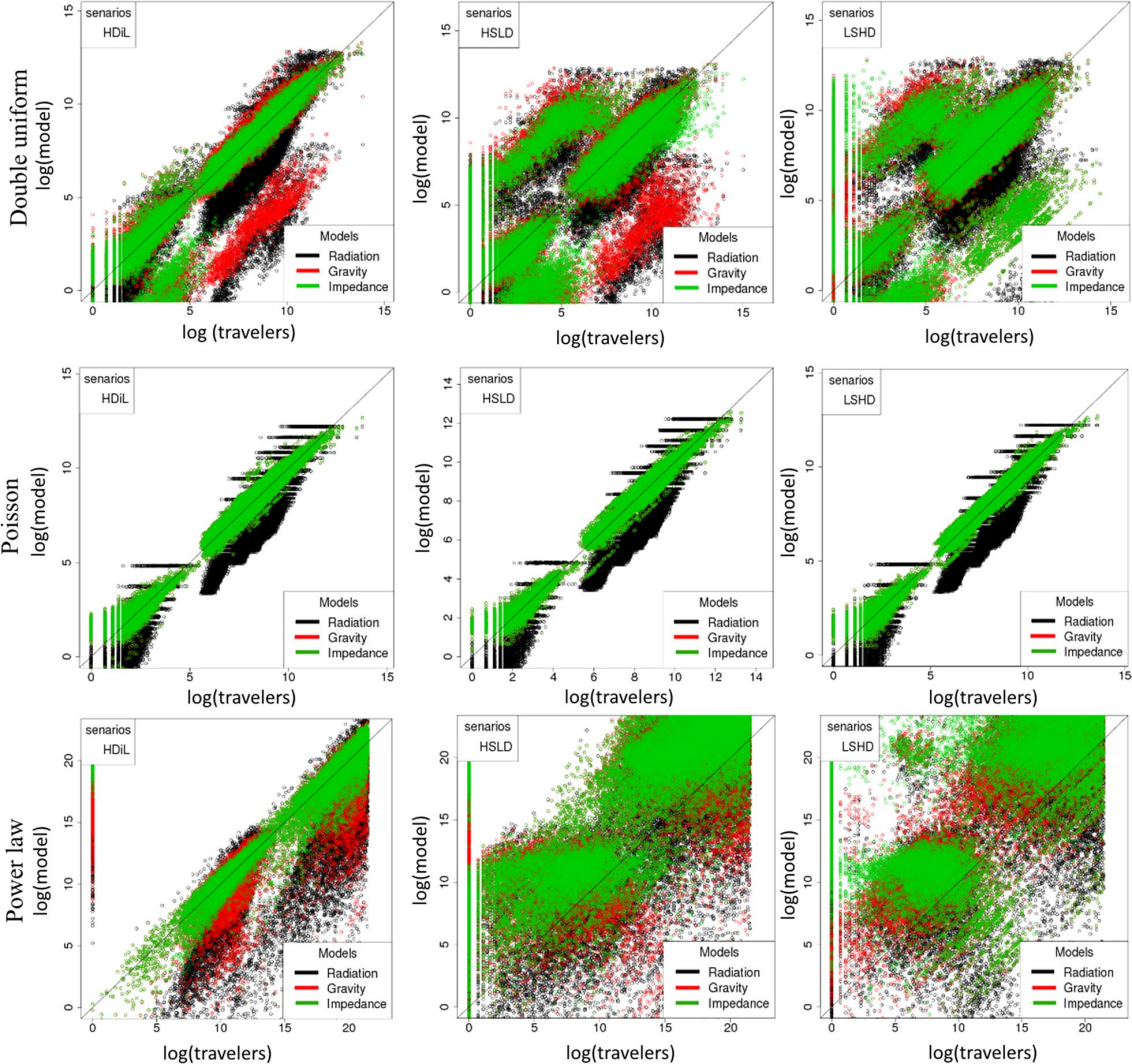
For each scenario separately, number of trips estimated by the three models versus simulated reference data. Number of simulations was limited to 160. Each dot represents the logarithm of the total number of trips on a given trajectory. Power law was simulated by taking the square of a selected range values

**Fig. 3:**
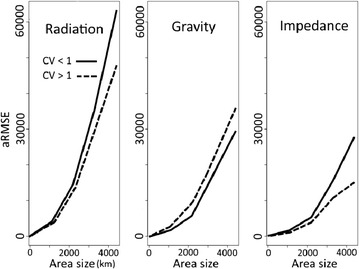
Variations in average root mean square error (aRMSE) according to area size. Plain lines and dotted lines correspond to groups of patterns with coefficients of variation of population sizes (CV) below and above 1, respectively

Similar tendencies were observed (both in aRMSE and bias) when using the grid distribution of locations, Poisson distribution or a power law and whatever the specific mobility scenarios: SPDD, LSDD or SLDD (Fig. 2). Assuming identical population sizes, the gravity and impedance models performed equally. Average error increased with area size. A hundred-fold increase in the standard deviation of population size (the coefficient of variation changing from 0.2 et 3) yielded a 5% aRMSE increase in the impedance model and 7 and 10% aRMSE decreases in the radiation and gravity models, respectively (Fig. 3). Thus, in heterogeneous settings, the impedance model produced the most accurate absolute flow estimates, as compared with older mathematical models.

**Fig. 4:**
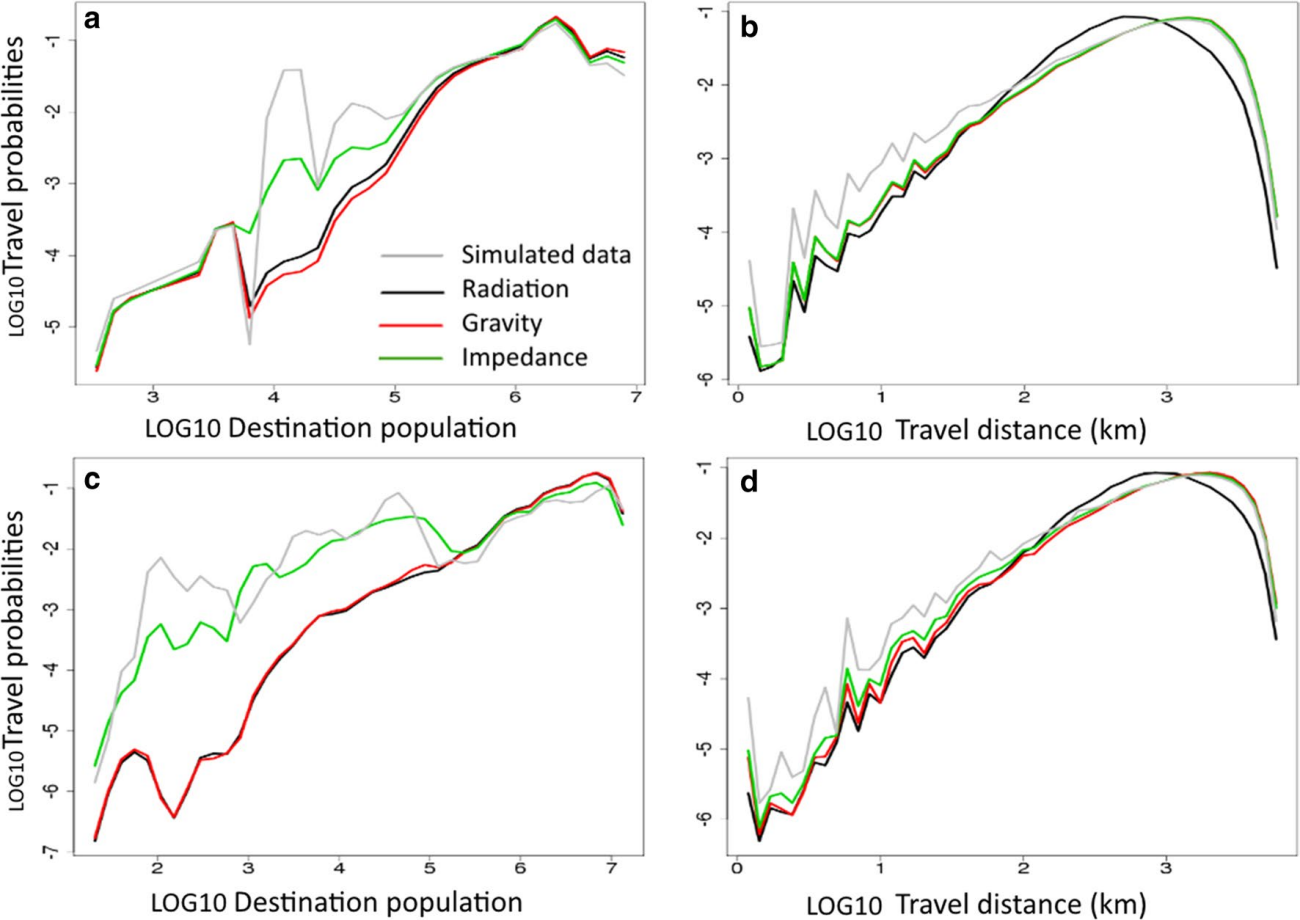
Probability of travel according to destination population, travel distance and population distributions. CV stands for the coefficient of variation of population sizes. Panels *a* and *b* stand for homogeneous population size (CV < 1) and panels *c* and *d* for heterogeneous population size (CV > 1). Short-distance mobility and mobility to destinations with small destinations are best predicted in heterogeneous patterns with the impedance model

Probabilities of mobility were estimated according to destination population, on the one hand, and according to travel distance, on the other. The predictions made with the impedance model were the closest to simulated data, regardless of population heterogeneity. When predicting short-distance mobility and mobility to destinations with small populations, the impedance model outperformed the gravity and radiation models, especially in the case of heterogeneous populations (Fig. 4).

#### Bias

**Fig. 5:**
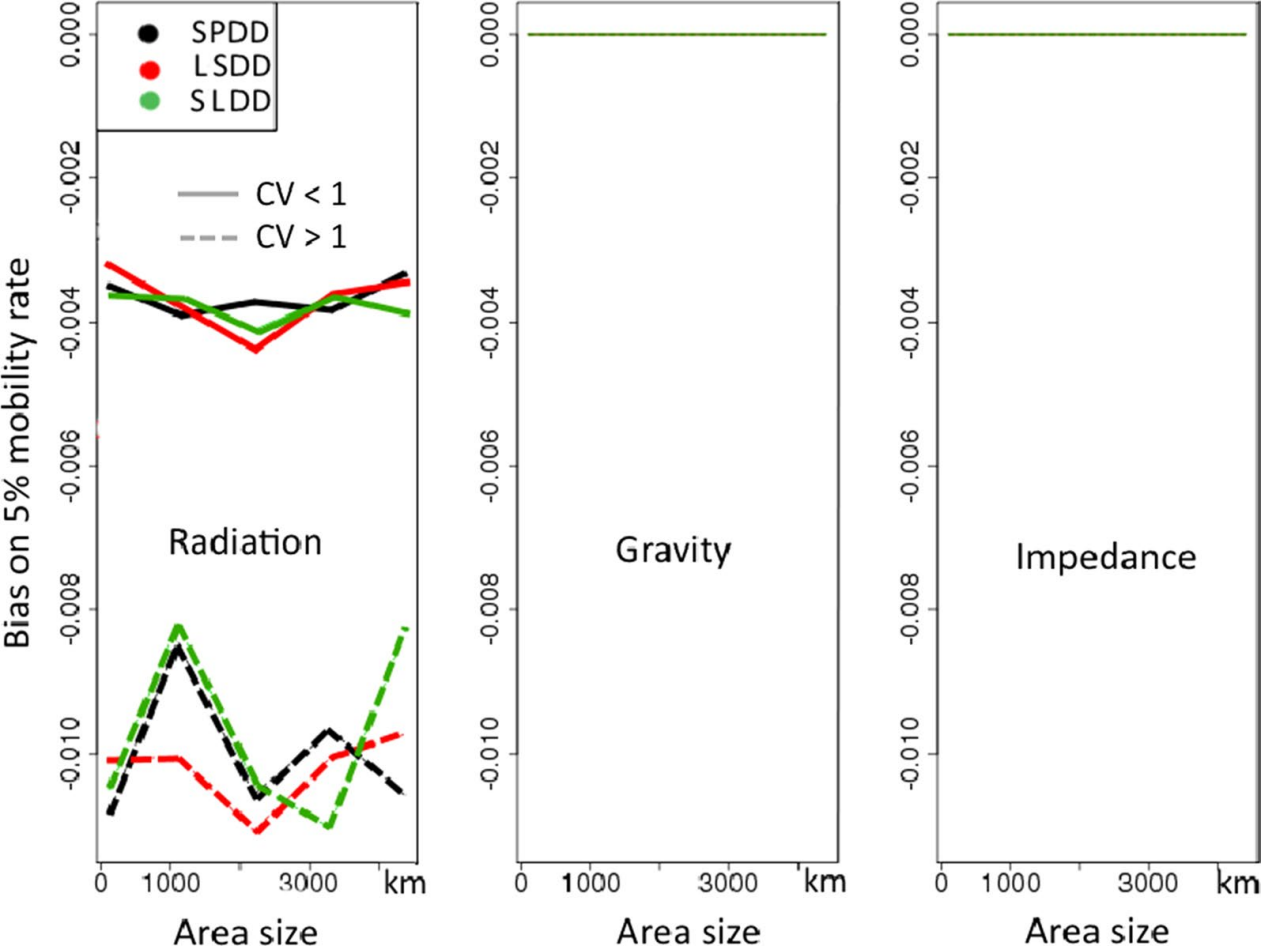
Bias according to model, area size, population distributions and scenarios of simulation. CV stands for the coefficient of variation of population sizes. SPDD: source population and distance deterrence, LSDD: large to small population with distance deterrence, SLDD: small to large population with distance deterrence

Bias in the 5% reference probability of mobility used to generate data is represented in Fig. 5 for various area sizes and for two levels of population heterogeneity.

The impedance and gravity models were unbiased regardless of the spatial pattern. The radiation model underestimated the overall probability of mobility. Bias was persistent regardless of the scenario and increased in the case of heterogeneous patterns: *δ* = − 0.0099 for heterogeneous populations versus − 0.0037 for homogeneous ones. Bias persisted in the grid distribution of locations.

#### Likelihood of the mobility scenarios

**Fig. 6:**
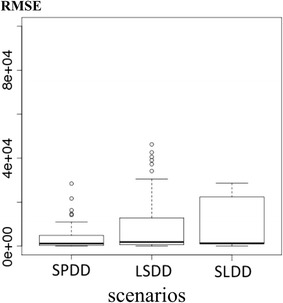
Average root mean square error over all simulations for each scenario. The SPDD scenario appears to be the most plausible one with regard to simulated data

Figure 6 summarizes the aRMSE estimates on the total number of trips in each scenario over five hundred simulated patterns. The SPDD scenario yielded the lowest aRMSE, making it a more plausible hypothesis for modeling human mobility (ANOVA test, *p* value = 0.03).

### Accuracy of the transmission model assuming various mobility patterns for the 2010 Haiti cholera epidemic

Statistical dispersion measures comparing population heterogeneity for both spatial definitions are presented below (Table 1). The coarse pattern (which includes 78 aggregated units) displays greater population heterogeneity.

**Table 1:** Statistical dispersion measures for two spatial definitions of the Haitian population

Measure	140 spatial units	78 spatial units
IQR	39,232	80,251
SD	103,511	309,417
CV	1.43	2.39

*IQR* interquartile range, *SD* standard deviation, *CV* coefficient of variation

 Transmission parameters estimated with the Metropolis–Hastings Markov Chain Monte Carlo algorithm using a CDRs-based fine-scale mobility matrix (*n* = 140) are presented in Table 2.

**Table 2:** Transmission parameters used in the basic SIR framework

Parameter	Units	Value	References
*β*	d^−1^	1 (0.96–1.16)	Fitted
*γ*	d^−1^	0.93 (0.89–0.97)	Fitted

*β* indicates the contact rate, which can be detailed as *β* = *pc*, where *p* denotes the probability of getting infected when coming into contact with an infected individual, and *c* is the per-capita contact rate; *γ* indicates the recovery rate

CDRs data were used to calibrate the transmission parameters. These parameters were then entered in new models that lacked mobility patterns.

Table 3 shows the AIC results obtained with different mobility matrixes, assuming that the rate of mobility from each location remains unknown. Both the matrix structure and the overall probability of mobility were tested. Results are shown for both spatial definitions. The CDRs-based model was taken as a reference when computing AIC variations. The impedance model performed well with heterogeneous spatial distributions, in which locations were defined as significant population agglomerations. Aggregated CDRs failed to fit the epidemiological data properly, even when using the most realistic parameters obtained with the fine-scale definition (AIC = 25,870 with the coarse definition vs. − 5200 with the fine-scale definition). Using the coarse definition, the AIC decreased by 22% for the impedance model, by 20% for the gravity model, and by 8.4% for the radiation model.

**Table 3:** Minimal AIC values, assuming that the overall probability of mobility (α) is to be fitted

Model	AIC *n* = 140	*α* *n* = 140	∆_*AIC (%)*_ *n* = 140	AIC *n* = 78	*α* *n* = 78	∆_*AIC (%)*_ *n* = 78
CDRs	− 5200	0.14		25,870	0.14	
IM	9000	0.01	+ 273	20,171	0.5	− 22
GM	16,185	0.01	+ 411	20,616	0.5	− 20
RM	9005	0.05	+ 273	23,696	0.09	− 8.4

AIC values obtained with each mobility model, for the 2010 Haiti cholera epidemic, assuming that the overall probability of mobility (*α*) is not given by CDRs, but fitted. The indicated models are: impedance model (IM), gravity model (GM), and radiation model (RM). Results are presented for the two spatial definitions. ∆_*AIC*_ corresponds to the variation from the optimal AIC value derived from the CDRs

Table 4 shows analogous results obtained by assuming that the overall probability of mobility (*α*) is provided by CDRs.

**Table 4:** Minimal AIC values, assuming that the overall probability of mobility (α) is known from CDRs

Model	AIC *n* = 140	*α* *n* = 140	∆_*AIC (%)*_ *n* = 140	AIC *n* = 78	*α* *n* = 78	∆_*AIC (%)*_ *n* = 78
CDRs	− 5200	0.14		28,992	0.14	
IM	22,798	0.14	+ 538	20,852	0.14	− 28
GM	22,667	0.14	+ 535	21,136	0.14	− 27
RM	18,915	0.14	+ 464	23,800	0.14	− 18

Overall probability of mobility (*α*) was derived from the CDRs. The indicated models are: impedance model (IM), gravity model (GM), and radiation model (RM). Results are presented for the two spatial definitions. ∆_*AIC*_ corresponds to the variation from the optimal AIC value derived from the CDRs

Additional file 1: B shows the sensitivity analysis associated with the results presented in Table 3. The latter correspond to the AIC variations that followed from variations in mobility rates.

## Discussion

In this article, we proposed a new, parameter-free and intuitive model for predicting human mobility in the context of data scarcity. We evaluated the performances of the impedance model through intensive simulation, and compared it to the (non parameter-free) gravity model and the (parameter-free) radiation model. Our results suggest that when the number of trips from each location is known (as assumed in the radiation model and as extracted from CDRs), the impedance model provides the best equation for predicting the distribution of travelers towards destinations, in aggregated patterns.

The inclusion of reactive-diffusion equations in dynamic models of infectious disease transmission is a method of determining the spread of an epidemic in the absence of mobility data. This method assumes that the movements of all individuals are stochastic, happening by continuous progression throughout the geographical space and therefore, each individual can potentially visit all the geographical locations. Depending on the context, this assumption can be inaccurate [2], even if additional parameters may adapt the implications [18]. Recent works have shown that human mobility is better represented by a specific spatial network [19–21]. Each Individual usually does not visit all the locations. This results in saturation in the rate of epidemic spread, whereas the classical diffusion hypothesis does not admit a limit to the diffusion speed when the mobility rate increases [2]. However, in the context of the Black Death outbreak model, Gaudart et al. [18] used a local viscosity parameter proportional to the altitude and human density, thus modeling the maximal diffusion velocity. This approach excluded low population density/mobility from the first wave of the epidemic.

Our study also uncovered the existence of intrinsic biases associated with CDRs, depending on the spatial definition used. In fact, we found that the most precious information that can be extracted from CDRs are the rates of mobility from the source location, not the probabilities of mobility to different destinations.

In addition, our study showed that the radiation model typically underestimates mobility, and can therefore yield inaccurate epidemiologic predictions, as has already been suggested [9, 22–24]. Our stimulation approach was more systematic than those used in previous studies, (including that of Masucci et al. [23]), as these have mainly relied on empirical data from specific countries.

We found that probability of mobility according to distance and to size of destination population—as measured in several studies [6, 9]—is a pooled measure that may mask errors in core flow estimates. Estimates of the probability of mobility beyond a given radius can be flawed because flows are pooled before probability is computed. In the context of infectious disease spread, raw flows are more relevant than average probabilities. Moreover, RMSE is a more relevant measure to assess the performance of mobility models in epidemiology because it is directly based on absolute number of trips. The most reliable model is expected to yield the smallest RMSE.

The scenarios defined for data generation did not reveal anything specific. However, their formulation did help to account for a wide range of mobility hypotheses. The second scenario (SLDD) seemed to correspond to mobility patterns during peak periods of activity in both industrialized and less industrialized countries, as well as to patterns of rural migration in less industrialized countries. The third scenario (LSDD) resembled mobility patterns in industrialized countries during holiday periods, as well as patterns of populations fleeing conflict zones. While these results were consistent across all three scenarios, it is obvious that mobility is driven by far more complex motivations at the individual level—which may explain the unpredictability of even the best model for any given area [25]. Moreover, we know that CDRs are unreliable due to the uneven distribution of cell phone towers and the heterogeneous penetration of devices across populations [26].

In this paper, we also compared results obtained from the new impedance model to those obtained from the two other more classical mobility models, based on real epidemiological data from the 2010 Haiti cholera epidemic. However, this epidemic was governed by complex factors that may require mechanistic modeling that is more spatially explicit [17]. Here we used a basic SIR framework that failed to account for water contamination. Also missing from this framework were the role of the Artibonite river in spreading the disease, the impact of hurricane Thomas (from 29 October to 7 November 2010), and rainfall data [27]. To attenuate such biases, the calibration span was restricted to a 12-week period during which meteorological and hydrolytic factors were presumed to be less critical.

Mathematical mobility models generally assume that the attractiveness of a location is correlated to the size of its population. However, this association is not always present, and, depending on the field, geographical barriers to mobility (such as high elevation, water bodies, etc.) and cultural resistance must be accounted for in the model formula without complicating it. While the most common data include population sets that are usually defined in administrative rather than demographic terms [28], mathematical models are more accurate when relying on demographic entities. Redistribution according to demography can therefore enhance the performance of all mathematical mobility models [28]. In our study, the impedance model performed well for patterns in which populations were aggregated beyond administrative constraints.

Future estimations of human mobility will likely increasingly rely on big data (such as high-resolution mobile network data or CDRs, social network data, etc.) [3], as these become available worldwide. However, when no real data is available on heterogeneous populations as is often the case in low-income countries, the impedance model can provide an unbiased, parameter-free, intuitive, and accurate framework for estimating human mobility for the purpose of controlling the spread of infectious diseases.

## Conclusions

While dealing with scarcity of real mobility data, and especially when the population distribution is heterogeneous, the proposed new impedance model provides most accurate estimates of human mobility at populational level. Its use can improve epidemiological forecasting when reliable mobility data sources are not available.

## Additional file

**Additional file 1.**Details on mathematical models and sensitivity analysis

## Abbreviations

AIC: Akaike’s information criterion
aRMSE: average root mean square error
CDRs: call detail records
CV: coefficient of variation
GM: gravity model
IM: impedance model
IQR: interquartile range
LSDD: large to small population with distance deterrence (mobility scenario)
RM: radiation model
RSS: residual sum of squares
SD: standard deviation
SIM: subscriber identity module
SIR: susceptible–infected–recovered
SLDD: small to large population with distance deterrence (mobility scenario)
SPDD: source population and distance deterrence (mobility scenario)
