# Supplementary material for: Mathematical models for predicting human mobility in the context of infectious disease spread: introducing the impedance model
Source: Int J Health Geogr. 2017 Nov 22;16:42. doi: 10.1186/s12942-017-0115-7 (PMC5700689; doi:10.1186/s12942-017-0115-7)
Supplement: Supplementary file 1 — Additional file 1.Details on mathematical models and sensitivity analysis [file 12942_2017_115_MOESM1_ESM.docx]

**Additional file**

**Mathematical models for predicting human mobility in the context of infectious disease spread: introducing the impedance model.**

Kankoé Sallah *^a,b*^*, Roch Giorgi *^a,c^*, Linus Bengtsson *^d,e^*, Xin Lu *^d,e,f^*, Erik Wetter *^e,g^*, Paul Adrien *^h^*, Stanislas Rebaudet *^i,j^*, Renaud Piarroux *^j^* and Jean Gaudart *^a,c^*

*^a^ Aix Marseille Univ, INSERM, IRD, SESSTIM, Sciences Economiques & Sociales de la Santé & Traitement de l’Information Médicale, Marseille, France.*

*^b^ Prospective et Coopération, Laboratoire d’Idées, Bureau d’Etudes Recherche, Marseille, France*

*^c^ APHM, Hôpital de la Timone, Service Biostatistique et Technologies de l’Information et de la Communication, Marseille, France*

*^d^* *Department of Public Health Sciences. Karolinska Institutet, Stockholm, Sweden*

*^e^ Flowminder Foundation, Stockholm, Sweden*

*^f^* *College of Information System and Management, National University of Defense Technology, Changsha, China*

*^g^* *Stockholm School of Economics, Stockholm, Sweden*

*^h^ Ministère de la Santé Publique et de la Population, DELR, Haiti*

*^i^ APHM, Direction de la Recherche et de l’Innovation, Marseille, France*

*^j^ Institut Pierre Louis d'Epidémiologie et de Santé Publique, UMR S 1136 INSERM, UPMC, France*

*Correspondence to: Kankoé Sallah, Aix Marseille Univ, SESSTIM, Sciences Economiques & Sociales de la Santé & Traitement de l’Information Médicale, F-13385, Marseille, France

E-mail: [kankoe.sallah@univ-amu.fr](file:///H:\10-Roch\A_Lire\kankoe.sallah@univ-amu.fr)

1. **Mathematical models of human mobility**

**A1. Gravity model**

The gravity model of human mobility was developed by analogy with the classical law of gravitation, back to the 18^th^ century. It has since been adapted to various domains, for instance, to forecast shipping volume [[1](#_ENREF_1)] or to predict the movement of persons [[2](#_ENREF_2), [3](#_ENREF_3)]. The gravity model estimates the number of trips *F_ij_,* between two geographical locations *i* and *j*, knowing their population sizes *P_i_* and *P_j_* and the distance between them, *d_ij_*, as

|  | $F_{ij}=k\frac{P_{i}^{n}P_{j}^{m}}{f_{\gamma}(d_{ij})}$ | (Eq. S1) |
| --- | --- | --- |

where $f_{\gamma}$ is the distance deterrence function (mobility decreases as distance increases). $f_{\gamma}$ is an exponential or power function whose shape can be controlled by a parameter *γ*. Parameters *n*, *m*, *γ* and *k* are constants estimated from the reference data.

The fitting process usually involves the linearization of Equation 1 along with regression techniques [[4](#_ENREF_4)]. In this study, we use the power-function-based gravity model because it has proved more accurate in estimating mobility at country level [[5](#_ENREF_5), [6](#_ENREF_6)] $f_{\gamma}(d_{ij})=\frac{1}{(d_{ij})^{\gamma}}$.

Calibration is necessary to estimate parameters *k*, *m*, *n* and *γ*. Thus, the probability of *π_ij_* commuting or migrating from *i* to *j,* where *j* represents any possible destination, was obtained from Equation S1 as:

|  | $\pi_{ij}=\frac{F_{ij}}{T_{i}}=\frac{k\frac{P_{i}^{n}P_{j}^{m}}{d_{ij}^{\gamma}}}{\sum_{i\neq j} k\frac{P_{i}^{n}P_{j}^{m}}{d_{ij}^{\gamma}}}=\frac{kP_{i}^{n}\frac{P_{j}^{m}}{d_{ij}^{\gamma}}}{\sum_{i\neq j} kP_{i}^{n}\frac{P_{j}^{m}}{d_{ij}^{\gamma}}}=\frac{\frac{P_{j}^{m}}{d_{ij}^{\gamma}}}{\sum_{i\neq j} \frac{P_{j}^{m}}{d_{ij}^{\gamma}}}$ | (Eq. S2) |
| --- | --- | --- |

where *T_i_* corresponds to the total number of trips from location *i*, and $F_{ij}$to the absolute number of trips from *i* to *j.*

Equation S2 represents the standardized form of the power-function-based gravity model, where the probability *π_ij_* depends on parameters *m* and *γ*.

Since parameter tuning is impossible in data-scarce contexts, we reduced Equation S2 to its simplest form, assuming $m=\gamma=1$. Note that Equation S2 is comparable to the radiation model, which serves to estimate the relative probabilities of mobility from source *i* to destination *j*, as detailed below.

**A2. Radiation model**

The radiation model was developed by analogy with the processes of emission and absorption studied by physical scientists. It is known that a particle is absorbed by the closest location whose absorbance is greater than its own absorption threshold [[7](#_ENREF_7)]. By analogy, if population density around a source location is low, people will have to travel long distances to access suitable jobs, supplies, or infrastructure. By contrast, if population density around a source location is high, people will not need to travel as far. The probability of commuting from *i* to *j* is given by

|  | $\pi_{ij}=\frac{P_{i}P_{j}}{\left(P_{i}+S_{ij}\right)\left(P_{i}+P_{j}+S_{ij}\right)}$ | (Eq. S3) |
| --- | --- | --- |

where *P_i_* is the source population, *P_j_* the destination population, and *S_ij_* the total population in the circle of radius *ij* centered at *i* (excluding the source and destination populations). This model is parameter-free.

*T_i_* is the total number of trips from location *i.* It must be available or estimated [[5](#_ENREF_5)] in order to derive the absolute flow $F_{ij}$ from *i* to *j* ($F_{ij}=\pi_{ij}\times T_{i}$).

1. **Sensitivity analysis**

**B1. Sensitivity analysis on the overall probability of mobility, calibrating the cholera transmission model on 140 locations**.

For each possible value of the overall probability of mobility, the Akaike Information Criteria is computed, according to the formula $AIC=2\theta+\eta\ln\left(\frac{RSS}{\eta}\right)$ where $\theta$ is the number of estimated parameters in the model, and $\eta=n_{u}\times n_{w}$ is the number of data points (with $n_{u}$ and $n_{w}$being the number of spatial units and the number of weeks since the start of the calibration period, respectively). The sum of the squares of the residuals between model estimates and epidemiological records is denoted by RSS.

|  |
| --- |
| Figure S1. AIC variations according to overall probability of mobility, calibrating the cholera transmission model on 140 locations (blue: CDRs; red: radiation model; green: impedance model; orange: gravity model). |
|  |
| **B2- Sensitivity analysis in coarse and heterogeneous population distribution (n=78)**  Sensitivity analysis on the overall probability of mobility, calibrating the cholera transmission model on 78 locations. AIC was computed as above (B1). |

|  |
| --- |
| Figure S2. AIC variations according to overall probability of mobility, using the best-fit transmission parameters in a coarse distribution (n=78) (blue: CDRs; red: radiation model; green: impedance model; orange: gravity model). |

**References**

1. Kaluza P, Koelzsch A, Gastner MT, Blasius B: **The complex network of global cargo ship movements**. *Journal of the Royal Society Interface* 2010, **7**(48):1093-1103.

2. Zipf GK: **The P1P2/D hypothesis: on the intercity movement of persons**. *AmSociol Rev* 1946, **11**:677–686.

3. Thiemann C, Theis F, Grady D, Brune R, Brockmann D: **The Structure of Borders in a Small World**. *PloS one* 2010, **5**(11).

4. Flowerdew R, Aitkin M: **A method of fitting the gravity model based on the Poisson distribution**. *J Regional Sci* 1982, **22**(2):191-202.

5. Simini F, Gonzalez MC, Maritan A, Barabasi AL: **A universal model for mobility and migration patterns**. *Nature* 2012, **484**(7392):96-100.

6. Chen Y: **The distance-decay function of geographical gravity model: Power law or exponential law?** *Chaos, Solitons & Fractals* 2015, **77**:174-189.

7. Kittel C MP: **Introduction to solid state physics**, vol. 4. New York: Wiley; 1986.
